# Substantial overlap between symptomatic and asymptomatic genitourinary microbiota states

**DOI:** 10.1186/s40168-021-01204-9

**Published:** 2022-01-17

**Authors:** Boahemaa Adu-Oppong, Robert Thänert, Meghan A. Wallace, Carey-Ann D. Burnham, Gautam Dantas

**Affiliations:** 1 The Edison Family Center for Genome Sciences and Systems Biology, Washington University School of Medicine, St. Louis, Missouri USA; 2 Department of Pathology and Immunology, Washington University School of Medicine, St. Louis, Missouri USA; 3 Division of Infectious Diseases, Washington University School of Medicine, St. Louis, MO USA; 4 Department of Pediatrics, Washington University School of Medicine, St. Louis, Missouri USA; 5 Department of Molecular Microbiology, Washington University School of Medicine, St. Louis, Missouri USA; 6 Department of Biomedical Engineering, Washington University, St. Louis, Missouri USA

**Keywords:** Genitourinary microbiome, Urinary tract infections, Clinical diagnostics, Dysbiosis

## Abstract

**Background:**

The lack of a definition of urinary microbiome health convolutes diagnosis of urinary tract infections (UTIs), especially when non-traditional uropathogens or paucity of bacteria are recovered from symptomatic patients in routine standard-of-care urine tests. Here, we used shotgun metagenomic sequencing to characterize the microbial composition of asymptomatic volunteers in a set of 30 longitudinally collected urine specimens. Using permutation tests, we established a range of asymptomatic microbiota states, and use these to contextualize the microbiota of 122 urine specimens collected from patients with suspected UTIs diagnostically categorized by standard-of-care urinalysis within that range. Finally, we used a standard-of-care culture protocol to evaluate the efficiency of culture-based recovery of the urinary microbiota.

**Results:**

The majority of genitourinary microbiota in individals suspected to have UTI overlapped with the spectrum of asymptomatic microbiota states. Longitudinal characterization of the genitourinary microbiome in urine specimens collected from asymptomatic volunteers revealed fluctuations of microbial functions and taxonomy over time. White blood cell counts from urinalysis suggested that urine specimens categorized as ‘insignificant’, ‘contaminated’, or ‘no-growth’ by conventional culture methods frequently showed signs of urinary tract inflammation, but this inflammation is not associated with genitourinary microbiota dysbiosis. Comparison of directly sequenced urine specimens with standard-of-care culturing confirmed that culture-based diagnosis biases genitourinary microbiota recovery towards the traditional uropathogens *Escherichia coli* and *Klebsiella pneumoniae*.

**Conclusion:**

Here, we utilize shotgun metagenomic sequencing to establish a baseline of asymptomatic genitourinary microbiota states. Using this baseline we establish substantial overlap between symptomatic and asymptomatic genitourinary microbiota states. Our results establish that bacterial presence alone does not explain the onset of clinical symptoms.

**Video Abstract**

**Supplementary Information:**

The online version contains supplementary material available at 10.1186/s40168-021-01204-9.

## Background

Urinary tract infections (UTIs) are one of the most pervasive urological disorders and a substantial yearly socioeconomic burden [1, 2]. Women are at a significantly higher risk to contract UTIs than men, with 60% of all women experiencing at least one episode in their lifetime [3]. With the increase in antimicrobial resistance in microbes frequently implicated in UTI, the selection and efficacy of empiric antimicrobial therapy is a growing challenge [4]. Inconclusive or incorrect microbiological diagnosis can select for antimicrobial resistance through the initiation of inappropriate or ineffective antibiotic therapies.

Standard clinical diagnosis, comprising of microbial culture, microscopy, and automated biochemical urinalysis [5–7], is optimized for the detection of traditional uropathogens like uropathogenic *E. coli* (UPEC). In outpatient settings, approximately 80% of UTIs are attributed to UPEC, while the remaining 20% are associated with bacteria such as *Proteus mirabilis*, *Klebsiella pneumoniae*, or *Acinetobacter* species [2, 8]. UTIs are diagnosed in cases where urinary symptoms coincide with urinary inflammation and uropathogen abundance that exceeds a threshold of clinical significance (with 10^5^ CFU/mL commonly used) [9]. Extensive resources have been allocated to the improvement of microbial diagnosis and development of consensus criteria for the initiation of antimicrobial treatment [10–12]. As a result, expanded quantitative urine culture (EQUC) has greatly improved recovery of uropathogenic bacteria [12, 13]. However, even with increased specificity, diagnostic cultures often remain negative or inconclusive and reports of clinical overtreatment of urinary disorders are frequent [14–17].

The lack of a definition of urinary microbiome health convolutes UTI diagnosis. For decades, the urinary tract has been considered sterile. Recently, culture-independent sequencing and enhanced culturing showed that even the asymptomatic urinary tract harbors diverse microbial communities [18, 19], which can be grouped into ‘urotypes’—distinct compositions of urinary microbes in individual patients [13]. Some studies implicate the urinary microbiome in urinary tract disorders, like urgency urinary incontinence [13]. Conversely, the microbiota inhabiting all sections of the genitourinary system may play a role in preventing ascension of invading uropathogen through the urinary tract into the bladder [2]. Similarly, dysbiotic shifts of the genitourinary microbiota may be associated with urinary disorders like UTIs. It has recently been proposed that urinary disorders should be considered as distinct states on a spectrum of urinary microbiome health [20]. This paradigm could help clinicians assess important clinical conditions, such as UTIs, asymptomatic bacteriuria, or urgency urinary incontinence, and evaluate the necessity of commonly prescribed antimicrobial treatments. Therefore, establishing a baseline state of urinary microbiome health has been a medical priority [13, 21–26].

Here, we use metagenomic shotgun sequencing to establish a baseline of asymptomatic genitourobiome fluctuations in healthy individuals. We use this baseline to contextualize the microbial composition of urinary specimens collected from patients presenting with lower urinary tract symptoms, clinically categorized as no growth, ‘insignificant’, ‘contaminated’, or culture-positive. We reveal that the majority of microbiota compositions characterized in specimens from patients suspected to have UTI overlaps with asymptomatic microbiota states of healthy individuals. Further, by comparing the results of standard-of-care urinalysis with sequencing results we demonstrate that taxonomic and genomic diversity of the urinary microbiota in specimens from patients suspected to have UTI is commonly underestimated by culture-based urinalysis.

## Methods

### Urine sample collection

Following IRB approval, remnant urine specimens, including midstream, catheterized, and urine of uncertain collection type, from de-identified patients who had urine cultures submitted as part of routine clinical care where the physician had concern for UTI were used in this study (Table 1, Table S1). Specimens were submitted to the Barnes-Jewish Hospital/Washington University School of Medicine in Saint Louis, Missouri, United States as part of routine clinical care and standard urinalysis were performed. All specimens from putative UTI patients were cultured using standard of care methods: 1 μL was plated to a MacConkey and sheep’s blood agar plates (Hardy Diagnostics) using a 1-μL calibrated loop and incubated at 35 °C in CO_2_ for 24 h. Suspect urine specimens were classified into one of four categories based on standard-of-care clinical procedures: (1) *‘culture-positive’*, if the specimen had significant growth of one or two uropathogens (*n* = 48), (2) *‘contaminated’*, 3 or more bacterial species growing in concentrations above threshold in standard-of-care clinical culture (10^5^) (*n* = 6), (3) *‘insignificant’*, < 10^5^ colony forming unites/mL present during culturing (*n* = 17) and (4) ‘*no growth’*, specimen had no visible signs of microorganism growth during culturing (*n* = 51). Patient age, sex, ethnicity, and urinalysis results, including white blood cell count (cells/hpf) were collected from the medical record.

**Table 1:** Cohort summary

Clinical variable	Asymptomatic (*n* = 10)	Positive (*n* = 48)	Contaminated (*n* = 6)	No growth (*n* = 51)	Insignificant (*n* = 17)
Age (years), mean (SD)	31.4 (8.77)	57.9 (21.4)	58.7 (20.3)	49.5 (17.8)	45.2 (22.4)
Gender, no. (%)					
Females	10 (100)	37 (77.1)	5 (83.3)	21 (41.2)	13 (76.5)
Males	0	11 (22.9)	1 (16.7)	30 (58.8)	4 (23.5)
Race, no. (%)					
Caucasian	9 (90)	28 (58.3)	3 (50)	33 (64.7)	8 (47.1)
Black	0	19 (39.6)	3 (50)	16 (31.3)	9 (52.9)
Asian	1 (10)			1 (2)	0
Not specified	0	1 (2.1)	0	1 (2)	0
Patient type, no. (%)					
Inpatient	0	16 (33.3)	3 (50)	12 (23.5)	4 (23.5)
Outpatient	10 (100)	31 (64.6)	3 (50)	38 (74.5)	13 (76.5)
Not specified	0	1 (2.1)	0 (0)	1 (2)	0
Department, no. (%)					
General medicine		12 (25)	3 (50)	21 (41.2)	5 (29.4)
Emergency medicine		9 (18.8)	0 (0)	7 (13.7)	0 (0)
Oncology		5 (11.8)	1 (16.7)	6 (11.8)	1 (5.9)
Gynecology		4 (8.3)	1 (16.7)	4 (7.8)	4 (23.5)
Urology		3 (6.3)	0 (0)	3 (5.9)	3 (17.6)
Other		8 (16.7)	0 (0)	8 (15.7))	1 (5.9)
Not specified		7 (14.6)	1 (16.7)	2 (3.9)	3 (17.6)
Type of urine specimen, no. (%)					
Catheter	0	3 (6.3)	0 (0)	9 (17.6)	0 (0)
Midstream urine	10 (100)	19 (39.6)	6 (100)	31 (60.8)	13 (76.5)
Not specified	0	26 (54.2)	0 (0)	11 (21.6)	4 (23.5)

Urine samples were collected from ten asymptomatic participants enrolled through the Women’s and Infants Health Specimen Consortium (WIHSC) following an assessment of the recent medical history and current medications (see Extended Data 1). Exclusion criteria were the presence of lower urinary tract symptoms and antibiotic exposure in the 14 days prior to enrollment. Informed consent was obtained from all patients. Three urine specimens were collected from each asymptomatic volunteer, on average the collections were 1.61 days apart (range 1–6 days). Sampling was approved by the Human Research Protection Office (approval number 201401115).

### Sample processing and sequencing

Culture-positive specimens and specimens from asymptomatic volunteers were re-plated to MacConkey and sheep’s blood agar plates (BAP, Hardy Diagnostics) and incubated for 24h at 35 °C in 5% CO_2_. Species identity of isolates was determined with the VITEK MALDI-TOF MS v2.0 knowledgebase (bioMerieux). Four milliliters of all urine specimens was used to isolate metagenomic DNA. DNA extraction for all specimen types (i.e., isolates, slurries, urines) was performed using the BiOstic Bacteremia DNA Isolation Kit (Mo-Bio). DNA was sheered to a target size range of approximately 500–600 bp using the Covaris E220 sonicator with the following settings: peak incident power, 140; duty cycle, 10%, cycles per burst 200; treatment time 75 seconds; temperature 7 °C; sample volume 130 μl. Sheared DNA was purified and concentrated using MinElute PCR Purification Kit (Qiagen), eluting in 20 μl pre-warmed nuclease-free H2O. Purified sheared DNA was then end-repaired and Illumina adapters were ligated. Sample specific barcodes were annealed using T4 DNA ligase (New England BioLabs). Samples were purified using a MinEluted PCR Purification Kit (Qiagen) and size-selected to a target range of 400–900 bp on a 1.5% agarose gel. Size-selected DNA was enriched using a 18-cycle PCR reaction: a 25-μl reaction volume was prepared containing 2 μl of purified DNA, 12.5 μl 2x Phusion HF Master Mix (New England BioLabs), 1 μl of 10 MM Illumina PCR Primer Mix (5′-AAT GAT ACG GCG ACC ACC GAG ATC TAC ACT CTT TCC CTA CAC GAC GCT CTT CCG ATC T-3′ and 5′-CAA GCA GAA GAC GGC ATA CGA GAT CGG TCT CGG CAT TCC TGC TGA ACC GCT CTT CCG ATC T-3′) and 9.5 μl of nuclease-free H2O. Amplified DNA was size-selected to a target range of 500 bp on a 1.5% agarose gel and samples were pooled at 10 nM for sequencing. Sequencing libraries for a subset of 54 specimens were prepared using the Illumina Nextera XT 40 protocol. Libraries of all samples were sequenced on the Illumina NextSeq 500 HighOutput platform (Illumina, 2 × 150 bp).

Prior to all downstream analysis, Illumina paired-end shotgun metagenomics sequence reads were binned by barcode, quality filtered using Trimmomatic v0.3.038 (java-Xms1024m-Xmx1024m-jar trimmomatic-0.33.jar PE-phred 33 ILLUMINACLIP:TruSeq3-PE-2.fa:2:30:10 LEADING:10 TRAILING:10 SLIDINGWINDOW:4:20 MINLEN:60) and filtered from human DNA using DeconSeq 39 and the v38 of the human genome (default parameters) [27, 28].

### Metagenome and statistical analysis

We determined the composition of the microbial communities of all specimens using MetaPhlAn2 [29]. Relative abundances extracted from MetaPhlAn2 were used in down-stream analyses. HUMAnN2 was utilized for metabolic pathway prediction [30] and pathway abundance profiles were used for analysis. Analysis were performed using the packages vegan, labdsv, ggpubr, rsample, purr, dplyr, devtools, pairwiseAdonis, and permute in R version 4.0.2. Bray-Curtis distances were calculated using the vegan package and visualized as PCoA plots via the ggplot2 package. Urotypes were identified via hierarchical clustering as previously described [13]. Briefly, between sample Bray-Curtis distance was used for hierarchical clustering using the hclust function in *R* (method=’average’) and urotypes were identified as cluster of highly similar composition in the resulting dendrogram. Kruskal-Wallis test was used to compare microbiota community metrics (richness and Shannon diversity) between all diagnostic categories and samples collected from asymptomatic volunteers. Pairwise Wilcoxon Test was used for post-hoc pairwise comparisons and *P*-values were corrected using the Bonferroni correction for multiple hypothesis testing. Pairwise PERMANOVA were performed using the pairwiseAdonis package in R. *P* values were corrected for multiple hypothesis testing using Bonferroni-correction.

A potential impact of clinical covariates on the urinary microbiota composition in symptomatic specimens was evaluated using a nested PERMANOVA design on Bray-Curtis dissimilarity values in the adonis2() function in the vegan package. The model formula took the form of: adonis2(Bray-Curtis dissimilarity~’clinical variable’, data=df, permutations=perm)

with permutations blocked in diagnostic category (setBlocks(perm)<-with(df, ‘diagnostic category)) and nperm = 999.

Taxa differentially abundant in specimens from distinct diagnostic categories were identified using LefSe [31]. *P* values < 0.05 were considered statistically significant.

The microbial composition in clinical specimens was compared to that of the range of asymptomatic genitourinary microbiota compositions using permutation tests (1000 iterations). One of the three available samples per asymptomatic volunteer was randomly selected to generate the range of asymptomatic microbiota compositions based on Bray-Curtis distance in each interation. Deviation of the microbiota composition in clinical specimens from the asymptomatic distribution was assessed by calculating the geometric distance of each sample to the centroid of the asymptomatic distribution determined by canonical analysis of principal coordinates (CAP). To comprehensively account for the range of asymptomatic microbiota states observed in the longitudinal dataset of the asymptomatic patients (Fig. 2), 1000 permutations were performed and the average distance for each clinical sample was used for analysis.

## Results

### Cohort description

Remnant urine samples collected from 122 patients experiencing urinary symptoms (62% female, median age 51.5 years, 60% Caucasian) were included in this study (Table 1). Sample from both outpatient and inpatient setting were included, and specimens included both catheterized as well as midstream urine and patients from all hospital departments (Table 1). Specimens were categorized via standard-of-care clinical urine culture as: (1) *‘culture-positive’*, growth of 1–2 uropathogenic species at ≥10^5^ colony forming units (CFU)/mL (*n* = 48), (2) *‘contaminated’*, growth of ≥ 3 bacterial species at ≥ 10^5^ CFU/mL (*n* = 6), (3) *‘insignificant’*, bacterial growth < 10^5^ CFU/mL (*n* = 17), and (4) *no growth*, no bacterial or fungal growth (*n* = 51). As UTIs are observed more frequently in women [2, 32], we recruited ten asymptomatic female volunteers with no clinical evidence of a UTI and without antibiotic exposure in the 2 weeks preceding study enrollment as a control group. Urine samples were collected from this group at three timepoints.

### Characterization of the genitourinary microbiota

**Fig. 1:**
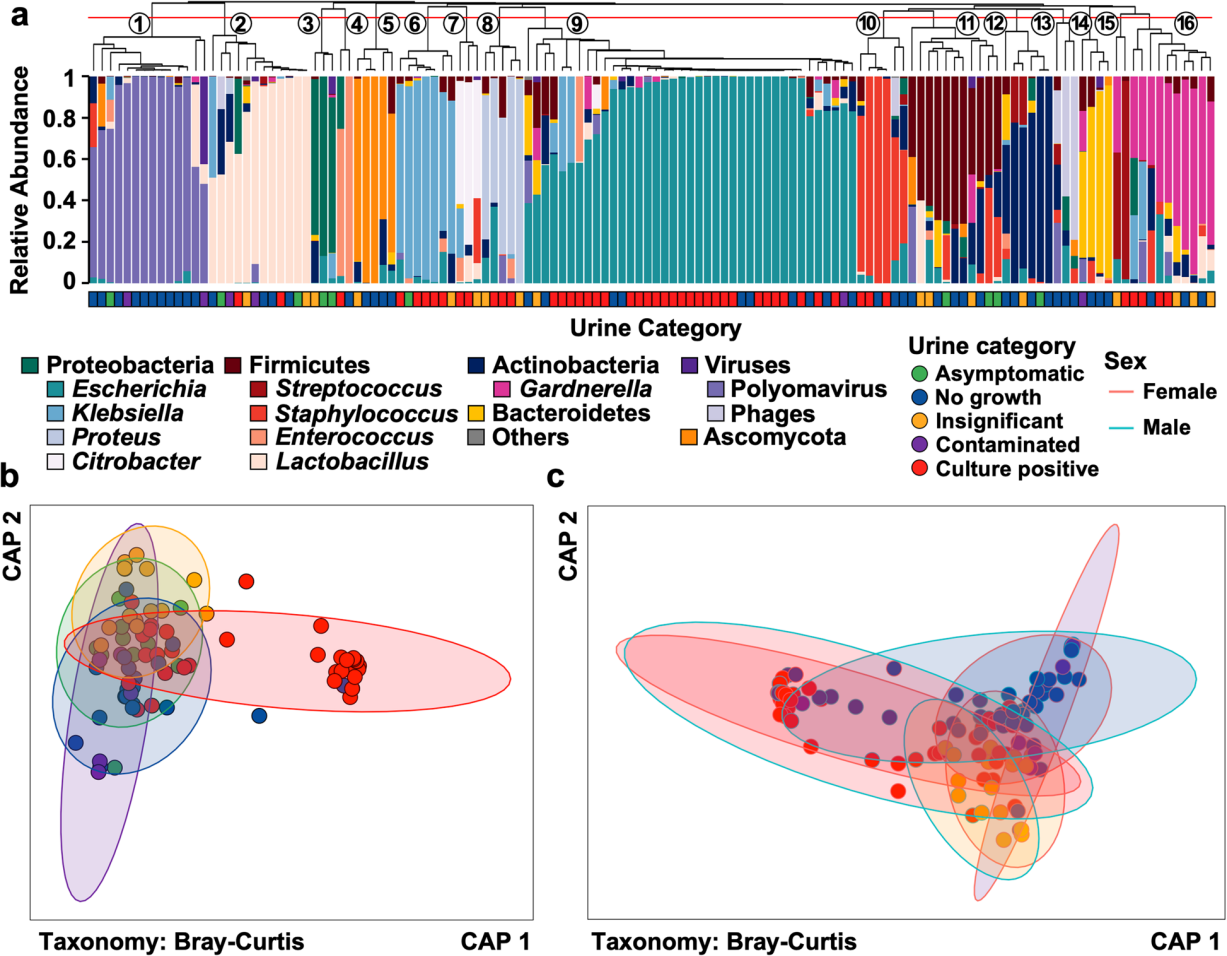
Microbiota composition varies between categories of diagnostic urinanalysis. **a** Microbiota composition in 122 suspected UTI urine specimens and 10 asymptomatic control samples clustered by relative abundance (hierarchical clustering based on Bray-Curtis distance). Clinical classification of each sample is depicted below the bargraph. Clusters of similar composition (urotypes) are indicated by numbers plotted onto the dendrogram. **b** Constrained analysis of principal coordinates based on microbiota composition of samples of all female participants. Individual samples are colored based on clinical categorization

Shotgun metagenomic sequencing showed that the genitourinary microbiota composition of clinical urine samples grouped into 16 distinct clusters (‘urotypes’) frequently predominated by a single bacterial taxon (Fig. 1a, Table S2). We observed a significant association between urotypes and diagnostic categories (*P* < 0.001, chi-square test). The majority of ‘culture-positive’ urine specimens were predominated by *Escherichia* spp. (Table 2). Predominance of other known uropathogens (*Klebsiella* spp., *Proteus* spp., *Citrobacter* spp.) was less common. The genitourinary microbiota of ‘no-growth’ specimens was predominated by common inhabitants of the genitourinary tract, specifically *Lactobacillus* spp., *Gardnerella* spp., or *Staphylococcus* spp., as well as in rarer cases members of the Actinobacteria (*Cutibacterium* spp., *Corynebacterium* spp.), Bacteroidetes (*Prevotella* spp.), or Firmicutes (*Ureaplasma* spp.) (Fig. 1a, Table 2). Notably, urotypes 1 and 5 were defined by high abundances of either viruses (Polyomavirus) or fungi (Ascomycota, predominately *Candida* spp.) and were frequently identified in ‘no-growth’ specimens (Table 2). Specimens classified as ‘insignificant’ or ‘contaminated’ lacked high relative abundances of established uropathogens (Table 2).

**Table 2:** Urotypes prevalence per diagnostic category of suspected UTI specimens

Urotype	Asymptomatic	Insignificant	No Growth	Contaminated	Culture-positive
**1 - Polyomavirus**	**10% (1/10)**	**–**	**21.57% (11/51)**	**33.33% (2/6)**	**–**
**2 -** ***Lactobacillus***	**20% (2/10)**	**11.76% (2/17)**	**7.84% (4/51)**	**33.33% (2/6)**	**4.17% (2/48)**
**3 - Proteobacteria**	**20% (2/10)**	**5.88% (1/17)**	**–**	**–**	**–**
**4 -** ***Enterococcus***	**–**	**–**	**1.96% (1/51)**	**–**	**2.08% (1/48)**
**5 - Ascomycota**	**–**	**5.88% (1/17)**	**7.84% (4/51)**	**–**	**–**
**6 -** ***Klebsiella***	**10% (1/10)**	**5.88% (1/17)**	**–**	**–**	**10.42% (5/48)**
**7 -** ***Citrobacter***	**–**	**5.88% (1/17)**	**–**	**–**	**4.17% (2/48)**
**8 -** ***Proteus***	**–**	**11.76% (2/17)**	**–**	**–**	**6.25% (3/48)**
**9 -** ***Escherichia***	**–**	**5.88% (1/17)**	**19.61% (10/51)**	**16.67% (1/6)**	**56.25% (27/48)**
**10 -** ***Staphylococcus***	**–**	**–**	**5.88% (3/51)**	**–**	**6.25% (3/48)**
**11 - Firmicutes**	**30% (3/10)**	**17.65 (3/17)**	**9.8% (5/51)**	**–**	**–**
**12 - Actinobacteria**	**10% (1/10)**	**5.88% (1/17)**	**7.84% (4/51)**	**–**	**–**
**13 - Phages**		**–**	**5.88% (3/51)**	**–**	**–**
**14 - Bacteroides**	**–**	**–**	**5.88% (3/51)**	**16.67% (1/6)**	**–**
**15 -** ***Streptococcus***	**–**	**5.88% (1/17)**	**–**	**–**	**2.08% (1/48)**
**16 -** ***Gardnerella***	**–**	**17.65 (3/17)**	**5.88% (3/51)**	**–**	**8.33% (4/48)**

To contextualize the genitourinary microbiota of the clinical categories within a range of asymptomatic urotypes, we characterized the similarity between the microbial compositions identified in clinical specimens and enrollment specimens collected from asymptomatic volunteers. Importantly, all asymptomatic volunteers were female, while the sex distribution in the clinical sample collection was heterogeneous (62% female). Therefore, we first assessed similarity only between asymptomatic females and the clinical specimens collected from female participants. Dimensionality reduction via constrained analysis of principal coordinates (CAP) indicated that the majority of clinical specimens cluster within the range of microbiota compositions observed in the asymptomatic group (Fig. 1b), with a subset of predominantly ‘culture-positive’ specimens forming a distinct cluster outside the main distribution. Subsequent comparison of the genitourinary microbiota of symptomatic women and men indicated no difference of the genitourinary microbiota composition in clinical specimens between sexes (Fig. 1c). This result was supported by permutational analysis of variance (PERMANOVA) indicating that independent of a sample’s clinical category, sex did not have a significant effect on microbiota composition of clinical specimens (*P* = 0.35, PERMANOVA). Similarly, race, patient type (inpatient, outpatient), and urine collection method did not explain a significant portion of the microbiota composition in specimens of symptomatic participant (*P* = 0.23, *P* = 0.76, and *P* = 0.41, respectively, PERMANOVA, Benjamini-Hochberg corrected), while hospital department and patient age significantly impacted the genitourinary microbiota composition in these specimens (*P* = 0.02, and *P* = 0.05, respectively, PERMANOVA, Benjamini-Hochberg corrected).

We observed no significant differences in microbial diversity between the genitourinary microbiota of asymptomatic and symptomatic women (*P* = 0.11, Kruskal-Wallis, Fig. S1a) or symptomatic women and men (*P* = 0.34, Kruskal-Wallis, Fig. S1b). Similarly, we found no significant differences in species richness between categories of female samples following *P* value correction (*P* > 0.05, Kruskal-Wallis with pairwise Wilcoxon post-hoc test, *P* value Bonferroni corrected, Fig. S1c) and richness did not differ between specimens collected from symptomatic men and women irrespective of clinical category (*P* = 0.09, Kruskal-Wallis, Fig. S1d). Functional characterization of the urinary microbiome using HUMAnN2 [30], highlighted a similar deviation of a set of ‘culture-positive’ urine specimens from the overlapping distribution of symptomatic and asymptomatic microbiome states (Fig. S2a). However, diagnostic urine category did not explain a significant amount of the variance in the functional microbiome composition for symptomatic vs asymptomatic women (*P* = 0.54, PERMANOVA). Moreover, the functional profile of the genitourinary microbiota did not differ between the samples of male and female symptomatic participant (*P* = 0.21, PERMANOVA, Fig. S2b).

### Temporal stability of the asymptomatic genitourinary microbiota

**Fig. 2:**
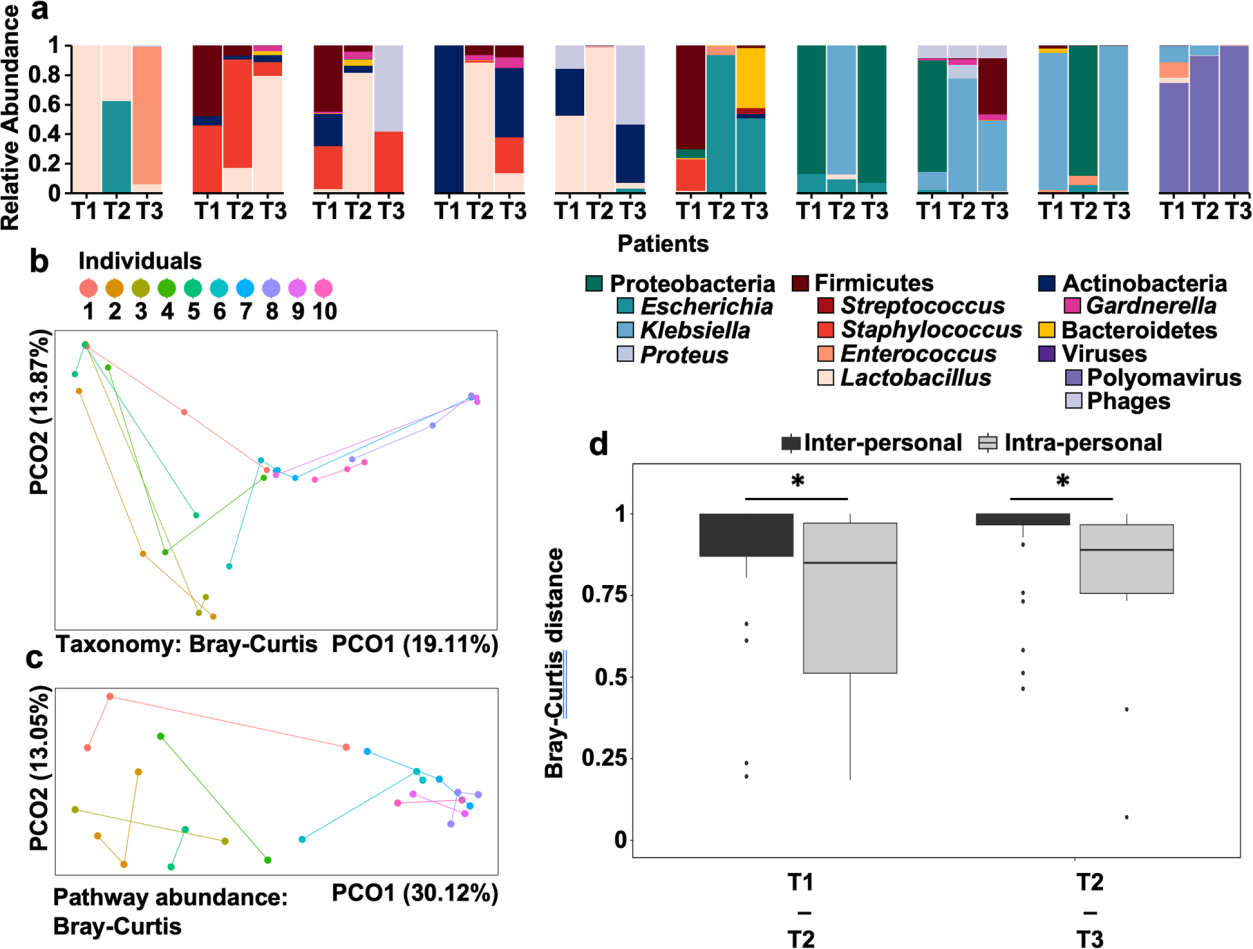
The genitourinary microbiota composition of asymptomatic patients fluctuates. **a** Relative abundance of microbiota in samples collected at three consecutive time points (T1, T2, T3) from 10 asymptomatic volunteers. **b** Principal coordinate analysis of the microbiota composition in the three urine specimens collected from 10 symptomatic volunteers (*n* = 30). Points are colored by patient and subsequently collected samples are collected via lines. **c** Principal coordinate analysis based on functional pathway abundance determined via HUMAnN2 in the longitudinal urine specimens collected from 10 symptomatic volunteers (*n* = 26). Four samples did not yield sufficient functional resolution and were excluded before analysis. Points are colored by patient and subsequently collected samples are collected via lines. **d** Bray-Curtis distance of the microbiota composition of subsequently collected urine specimens from asymptomatic volunteers (intra-personal) compared to the distance between patients collected from different individuals at the same timepoint (inter-personal). Bray-Curtis distances between groups for each interval were compared using the Wilcoxon signed-rank test with *P < 0.05

To characterize the range and temporal stability of asymptomatic genitourinary microbiota states in healthy individuals, we analyzed the microbiota composition in two additional urine specimens collected at subsequent time points per asymptomatic volunteer (average distance between samplings 1.61 days (range 1–6 days)). We observed remarkable compositional and functional shifts of the genitourinary microbiome for the majority of investigated individuals (Fig. 2a–c). In 9/10 asymptomatic individuals, the dominant bacterial taxon shifted between consecutive timepoints, often to taxa not detected in preceding samples (Fig. 2a, Table S2). These shifts were frequently associated with increased abundances of bacterial taxa with known uropathogenic potential like *Klebsiella* (*K. pneumoniae*), *Escherichia* (*E. coli*), *Enterococcus* (*E*. *faecalis*), or *Enterobacter (E. cloacae*) (Fig. 2a, Table S2) and highlight the transient nature of microbial colonization of the genitourinary area.

While the microbial composition of urine specimens collected at different timepoints from the same individual was variable, characterization of the intra- and interpersonal Bray-Curtis distance revealed a detectable person-specific genitourinary microbiota signature (Fig. 2d). The Bray-Curtis distance of consecutive urine specimens from the same individual was significantly lower compared to the between-patient distance at both investigated timepoints (*P* = 0.03 and *P* = 0.003, respectively, Wilcoxon rank-sum test).

### Diagnostic categories reflect different states of urobiome health

**Fig. 3:**
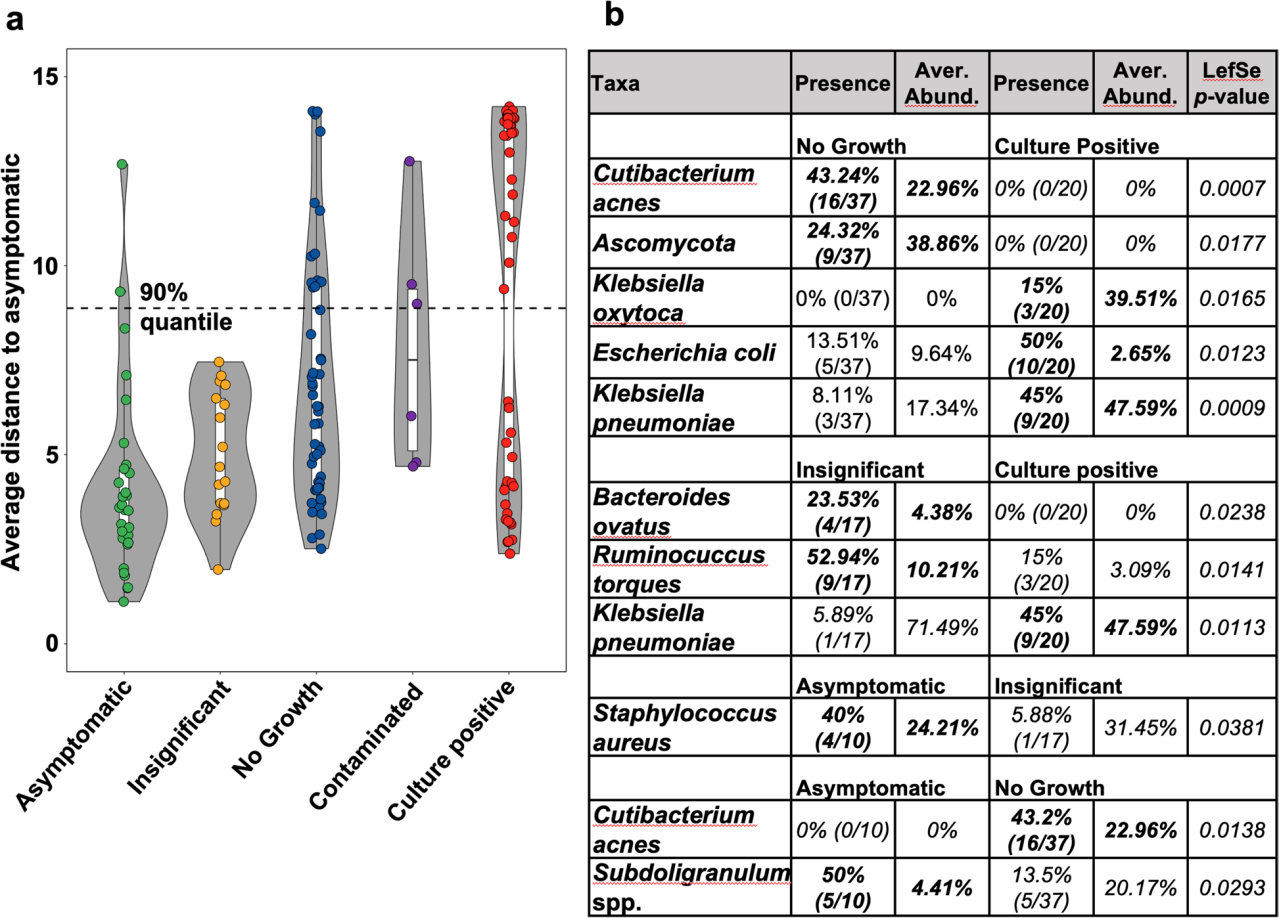
The microbiota composition of diagnostic categories correspond to different states of genitourinary microbiota health. **a** Distance of the microbiota composition determined in urine sample classified into different diagnostic categories to the centroid of the distribution of the distribution of asymptomatic microbiota states determined by constrained analysis of principal coordinates in 100 iterations including all longitudinally collected specimens. Dotted line marks the 90% quantile of distances for specimens collected from the asymptomatic population. **b** Significantly enriched microbiota in suspected UTI specimens from different clinical categories. Significance of enrichment was determined via linear discriminatory analysis as implemented in LefSe with a significance threshold of *P* < 0.05

To place the microbiota composition of suspected UTI urine specimens within the observed range of genitourinary microbiome health, we determined how clinical specimens deviate from the composition of the microbiota in urine samples collected from asymptomatic volunteers using permutation tests (see “Methods” section). The microbial compositions of specimens categorized as ‘insignificant’ and ‘no-growth’ predominantly fall within the 90% quantile of the asymptomatic reference group (Fig. 3a). Specimens with high relative *Escherichia* spp. abundances in ‘culture-positive’ specimens deviated from the distribution of asymptomatic states, while other bacteria with uropathogenic potential like *Klebsiella* or *Proteus* are better represented in the range of asymptomatic microbiota states (Fig. S3a).

To identify genitourinary microbiota potentially associated with clinical symptoms in absence of genitourinary microbiome dysbiosis, we performed enrichment analysis using LefSe [31] on all samples within the 90% quantile of the asymptomatic distribution. We found no significant enrichment of bacterial taxa in ‘culture-positive’ specimens when compared to the enrollment specimens of asymptomatic volunteers (Table S3). However, bacteria with uropathogenic potential (*Klebsiella pneumoniae*, *Klebsiella oxytoca*, and *Escherichia coli*) were overrepresented in ‘culture-positive’ specimens when compared to ‘no-growth’ samples, which were in turn enriched for *Cutibacterium acnes* and Ascomycota (Fig. 3b), indicating that cultivability of microorganisms in standard-of-care diagnostics is the main determinant of clinical classification between these two categories. The enrichment of anaerobic bacterial taxa (*Ruminococcus torques*, *Bacteroides ovatus*) in specimens classified as ‘insignificant’ when compared to ‘culture-positive’ samples further indicates that cultivability of organisms limits diagnostic sensitivity of conventional standard-of-care culture-based urine analysis.

To identify a potential relationship between urinary symptoms and genitourinary microbiome dysbiosis, we analyzed available white blood cell count data, commonly used as a clinical indicator of urinary tract inflammation (Fig. S3b). In the majority of suspected UTIs classified as ‘insignificant’ (8/10, 80%) and a subset of cases classified as ‘no-growth’ (14/39, 35.9%), white blood cell counts (WBC) were found to be above the clinical threshold for urinary inflammation (WBC ≥ 5 per high power field). Clinical samples with indication for urinary tract inflammation were evenly distributed between samples from within and outside the 90% quantile of the distribution of asymptomatic urinary microbiome compositions (Figs. S3b and S4), indicating that genitourinary microbiome dysbiosis alone does not explain the onset of urinary symptoms.

### Diagnostic culturing can fail to reflect microbial composition of urine specimens

Culture-based recovery of the genitourinary microbiota from urine specimens represents a keystone of routine medical urine diagnostics. Therefore, we determined the ability of standard-of-care diagnostic culturing to comprehensively recover the genitourinary microbiota. We sequenced culture slurries of ‘culture-positive’ specimens and compared the recovered microbiota composition to directly sequenced urine. While microbiota compositions of culture slurries and directly sequenced urine were generally similar, a subset of samples exhibited high compositional differences between sample types (Fig. S5a). In these specimens, single bacterial taxa representing only a fraction of the microbiota composition identified by direct urinary sequencing were selectively enriched under culture conditions (Fig. S5b).

**Fig. 4:**
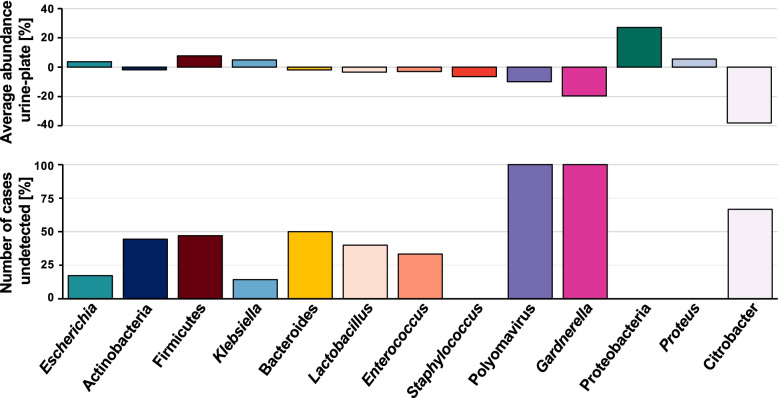
Modified standard-of-care urine culture misses bacteria with uropathogenic potential. Top panel—average enrichment of distinct taxa in cultured urine (positive values) or directly sequenced urine specimens (negative values). Bottom panel—percentage of cases in which taxa were detected in metagenomic sequencing of urine specimens but went undetected in culture of urine specimens

Specifically, *Gardnerella* spp. (undetected in 6/6 specimens) and Polyomavirus (6/6), a virus that would not be recovered in traditional culture, were systematic underrepresentated in culture-based uropathogen detection (Fig. 4). Similarly, culture-based methods failed to detect *Citrobacter freundii*, an emerging healthcare-associated urinary pathogen [33], in 2 of 3 cases and underestimated its abundance when detected (Fig. 4). While culture-based detection of potential uropathogens like *Klebsiella* (undetected in 2/13) or *Escherichia* (undetected in 5/28) was more reliable, these results indicate that culture-based microbial determination does not reflect the composition of diagnostic urine specimens.

## Discussion

Following the discovery of the urinary microbiome, six characteristic genitourinary microbiota compositions have been identified in a cohort of patients with urgency urinary disorder and asymptomatic controls [13]. It has been proposed that urinary tract disorders represent states of dysbiosis on a spectrum of urinary microbiome health [20]. However, studies of the range and temporal dynamics of asymptomatic urinary microbiota states are scarce [24] and more data is needed to assess the urinary dysbiosis hypothesis. Here, we analyze the microbial composition of suspected UTI urine specimens and contextualizing them within the spectrum and fluctuations of asymptomatic genitourinary microbiota states of healthy female volunteers.

We observed that previously described genitourinary microbiota compositions [13], predominated by *Lactobacillus*, *Gardnerella*, Enterobacteriales*,* or *Staphylococcus*, can be found in a large subset of suspected UTI urine specimens. Our results further confirm that less prominent taxa, including Polyomavirus [34, 35], Ascomycota (*Candida* spp.) [36], and, less frequently, *Cutibacterium* spp., *Corynebacterium* spp., *Prevotella* spp., or *Ureaplasma* spp. [13, 37, 38], can dominate symptomatic urine specimens. We find that specimens classified as ‘culture-positive’ are frequently predominated by *Escherichia*, confirming previous results showing that, as intended [39], standard-of-care clinical urinalysis preferentially recovers organisms considered as the main etiological agents of urinary disorders [18, 40]. The spectrum of urotypes recovered from specimens classified as ‘no-growth’ or ‘insignificant’ included microorganisms impossible to recover via standard-of-care urine culturing (Polyomavirus) or not considered uropathogenic (*Lactobacillus* spp., *Prevotella* spp., *Cutibacterium acnes*).

To determine whether genitourinary microbiota compositions identified in suspected UTI specimens reflect different states of genitourinary microbiota dysbiosis, we analyzed the microbial composition of urine specimens collected from asymptomatic volunteers. As previously reported [13, 21–26], we observed high inter-individual differences of the urinary microbiota composition, highlighting the lack of a single ‘asymptomatic’ urobiome composition associated with asymptomatic states. Notably, urotypes identified in asymptomatic volunteers (Polyomavirus, *Staphylococcus aureus*, *Klebsiella pneumoniae*) overlapped with those observed in clinical specimens collected from patients with urinary symptoms, indicating that strain differences or host factors may determine onset of clinical symptoms. Characterization of the temporal stability of urotypes revealed fluctuations in the bacterial and functional compositions of person-specific urobiomes (Fig. 2a). This observation is consistent with a recent study that combined 16S rRNA sequencing and culturing of daily urine specimens collected from asymptomatic young women to show that the genitourinary microbiota fluctuates within days between distinct community states, impacted by factors including menstruation and vaginal sex [24]. While specimens longitudinally collected from the same person were significantly more similar to one another than to samples from unrelated individuals (Fig. 2d), the stability of an individual’s genitourobiome was substantially lower than what has been reported for other body sites [41, 42]. The intrapersonal fluctuations of the genitourinary microbiota highlights the urinary tract as an area of frequently changing microbial stimuli at the interface with human immunity. Dramatic microbiome shifts are considered strong inflammatory stimuli and have been associated with disease flares in diverse body habitats [43, 44]. Consistent with recent reports [24], we observe that female asymptomatic subjects frequently shift between urotypes, potentially caused by blooms of individual taxa [45, 46]. Future studies should investigate the immunostimulatory effects of these fluctuations.

The longitudinal sampling of asymptomatic volunteers allowed us to investigate whether urinary symptoms of patients with suspected UTIs are associated with urinary dysbiosis or represent phases of heightened urinary sensitivity. ‘Culture-positive’ specimens predominated by *Escherichia coli* fell outside the 90% quantile of asymptomatic microbiota states. Importantly, previous studies have shown that *E. coli* is a relatively common member of the genitourinary microbiota and can be present at high abundance in asymptomatic women [24, 40]. Our results, however, indicate that *E. coli* is over-represented in symptomatic specimens compared to asymptomatic controls, consistent with its role as the dominant uropathogen [47]. The microbiota composition of all specimens categorized as ‘insignificant’, the majority of ‘no growth’, and a subset of ‘culture-positive’ urines were well-represented within the 90% quantile of asymptomatic microbiota states. This observation confirms results of prior studies, which have shown significant overlap of asymptomatic and symptomatic urobiome states of patients with urinary disorders like urgency urinary incontinence [13] or recurrent UTIs [46]. The overlap of urotypes and species between asymptomatic patients and suspected UTI cases may indicate that immune responsiveness, rather than bacterial presence alone, determines the onset of urinary symptoms. Indeed, we show variable genitourinary inflammation (white blood cell count ≥ 5) in urines categorized as ‘contaminated’, ‘insignificant’ and ‘no-growth’ (Fig. S3), for which the genitourinary microbiota compositions falls into the range of asymptomatic microbiome states. A model of UTI in which inflammatory responsiveness to bacterial colonization of the urinary tract determines the occurrence of urinary symptoms is consistent with the observation that high urine titers of potentially uropathogenic bacteria are not inevitably associated with urinary symptoms [48].

Bacterial recovery from urine specimens and resulting bacteriological thresholds are of exceptional importance for the diagnosis of urinary disorders and have been controversial [49–51]. One challenge with this approach is the binary nature of a specific cut-off for “positive” vs. “negative” results, understanding that like most biological pnenomenon there is a contininuum of findings and clinical correlates. Comparison of microbiota compositions of directly sequenced ‘culture-positive’ urine specimens with culture slurries indicated effective recovery of uropathogenic *Escherichia*, *Klebsiella*, and *Proteus* in culture-based urinalysis. However, bacteria traditionally not considered uropathogenic (*Gardnerella*, *Lactobacillus*, Polyomavirus) or less common uropathogens (*Citrobacter*, *Enterococcus*) frequently went undetected or were underrepresented in culture-based assessment (Fig. 4). Similar to previous studies [12, 18], our results highlight the limitations of standard microbial culturing for genitourinary microbiota recovery. While enhanced culturing protocols have been established to address these shortcomings, the clinical interpretive criteria for these methods are unresolved and modified versions of the standard urine culture are still widely used. Our results advocate for the general adoption of enhanced urine culture in some patient populations and highlight the need to study the role of microbes traditionally not considered uropathogenic in the pathogenesis of urinary disorders, which dominate the urinary tract in a large subset of symptomatic and ‘culture-positive’ specimens (Table S2, Fig. 4).

A limitation of our study was the availability of urine specimens collected from asymptomatic volunteers came exclusively from female volunteers and that limited clinical data are available on study subjects. Importantly, data on menstruation and vaginal sex, factors recently shown to significantly impact the genitourinary microbiota of asymptomatic women [24], were unavailable in the current study. Moreover, while we did not observe a significant impact of sex on urinary microbiota composition in suspected UTI specimens, studies have shown that key members of the female asymptomatic urinary microbiota (e.g., members of the genera *Lactobacillus*, *Gardnerella*) are absent in men [52, 53]. Therefore, it remains unanswered whether rapid species turnover observed in the urinary microbiome of asymptomatic female volunteers can similarly be observed in asymptomatic males.

## Conclusions

Collectively, our data establishes overlap between symptomatic and asymptomatic genitourinary microbiota states, highlighting that immune responsiveness, rather than bacterial presence, may determine the onset of clinical symptoms and determine genitourinary microbiome health. Prior work at the interface of bladder immunity and bacterial pathogenesis has indicated individualized sensitivities to recurrence of infectious episodes, which could explain how the same bacterial composition can result in different symptomatic outcomes [54]. Characterization of the mechanism that govern individualized sensitivities for developing urinary symptoms promises to move clinical diagnostics of urinary disorders beyond the determination of bacterial presence, guide intervention strategies and establish a more comprehensive picture of what constitutes urinary microbiome health.

## Supplementary Information

**Additional file 2: Figure S1.** Community metrics of urine specimens from distinct diagnostic categories and asymptomatic volunteers do not differ. a) and b), Shannon diversity of a) all female participant grouped by diagnostic categories and b) all symptomatic participants of both sexes. c) and d), microbiota richness of c) all female participant grouped by diagnostic categories and d) all symptomatic participants of both sexes. Individual samples are colored based on clinical categorization.

**Additional file 3: Figure S2.** Functional profile of the genitourinary microbiota. Principal coordinate analysis based on functional pathway abundance determined via HUMAnN2 for a) all female participants and b) all symptomatic participants of both sexes. Individual samples are colored based on clinical categorization.

**Additional file 4: Figure S3.** Samples outside the range of asymptomatic microbiome states are dominate by *E. coli* but not associated with increased inflammation. B) Scatterplot of the relative abundance of the most abundant intestinal microbiota from ‘culture-positive’ specimens plotted against each specimen’s average distance from the centroid of the asymptomatic distribution of microbiota states as depicted in Fig. 3a. Sample density at each distance is plotted on top of the graph. b) White blood cell count (cells/high power field - hpf) determined via microscopic examination in urine specimens classified into different diagnostic categories. Specimens are labeled based on whether the determined microbiota compositions fell within (dark bar) or outside (light bar) the 90% quantile of asymptomatic microbiota compositions as depicted in Fig. 3a.

**Additional file 5: Figure S4**. White blood cell count is independent of microbiota composition in the subset of investigate urine specimens. Microbiota composition of urine specimens (stacked bargraph) is grouped by clinical diagnostic category. Specimens are further grouped based on whether the determined microbiota compositions fell within (dark top bar) or outside (light top bar) the 90% quantile of asymptomatic microbiota compositions. Corresponding white blood cell counts determined via high power field microscopy are depicted in tiles under each bar.

**Additional file 6: Figure S5**. Microbiota states of individual specimens is not represented by standard-of-care urine culture. a) Bray Curtis similarity (1-Bray Curtis distance) of directly sequenced and cultured urine specimens. b) Stacked barchart depicting the microbiota composition of directly sequenced (left bar) and cultured (right bar) ‘culture-positive’ urine specimens. Pairwise Bray Curtis similarity is indicated in tiles below each sample pair.

**Additional file 7: Table S1.** Cohort metadata.

**Additional file 8: Table S2.** Taxonomic composition of urine metagenomes as determined via Metaphlan2

**Additional file 9: Table S3.** LefSe enrichment results

**Additional file 10.** Extended Data 1 Case report form asymptomatic participants

## Data Availability

The sequencing data supporting these studies conclusions has been uploaded to NCBI SRA under the BioProject accession number PRJNA700071. All other supporting information is available from the corresponding author upon request.

## Abbreviations

BAP: Blood agar plates
CAP: Constrained analysis of principal coordinates
CFU: Colony forming units
EQUC: Expanded quantitative urine culture
UPEC: Uropathogenic *Escherichia coli*
UTI: Urinary tract infections
PERMANOVA: Permutational analysis of variance
WBC: White blood cell counts
WIHSC: Women’s and Infants Health Specimen Consortium
