# Supplementary figures and images for: Substantial overlap between symptomatic and asymptomatic genitourinary microbiota states
Source: Microbiome. 2022 Jan 17;10:6. doi: 10.1186/s40168-021-01204-9 (PMC8762997; doi:10.1186/s40168-021-01204-9)

**a**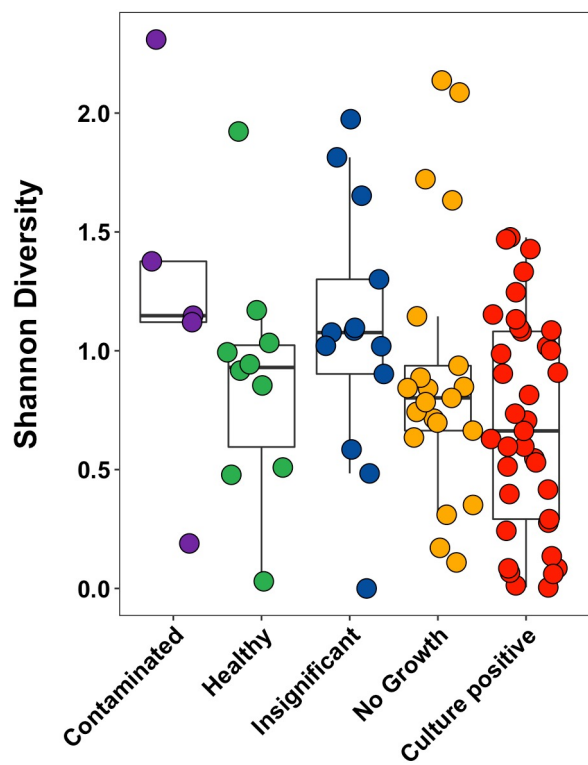**b**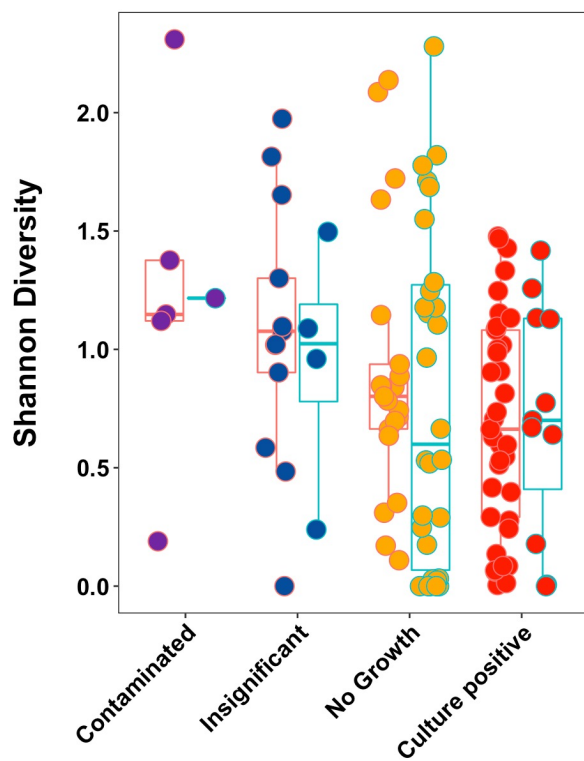**c**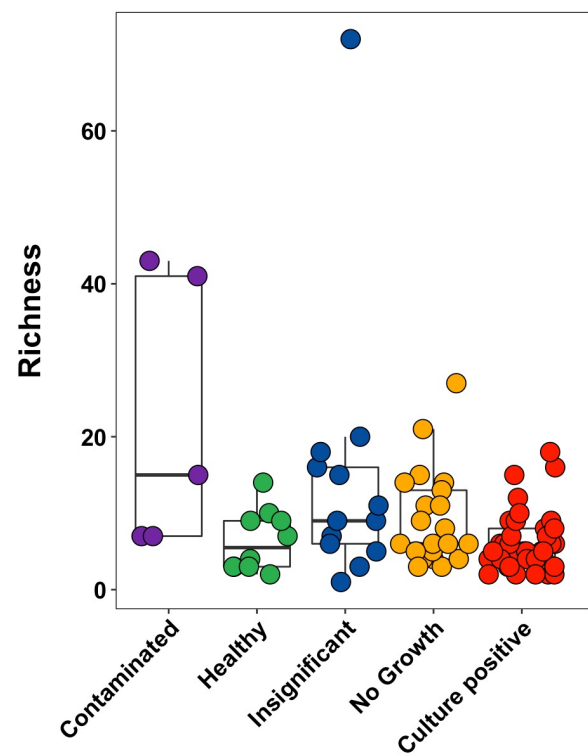**d**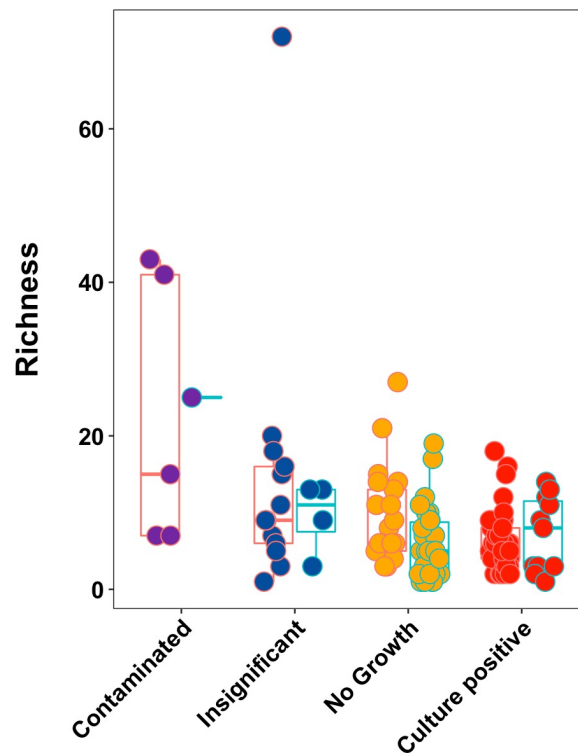

● No Growth  
● Insignificant  
● Contaminated  
● Culture positive

□ F  
□ M

Supplement: Supplementary file 2 — Additional file 2: Figure S1. Community metrics of urine specimens from distinct diagnostic categories and asymptomatic volunteers do not differ. a) and b), Shannon diversity of a) all female participant grouped by diagnostic categories and b) all symptomatic participants of both sexes. c) and d), microbiota richness of c) all female participant grouped by diagnostic categories and d) all symptomatic participants of both sexes. Individual samples are colored based on clinical categorization. [file 40168_2021_1204_MOESM2_ESM.pdf]

**a**

PCO 2

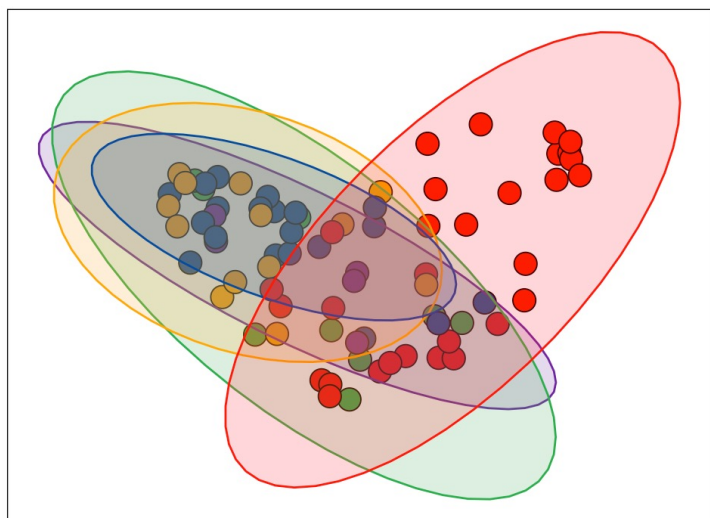

Functions: Bray-Curtis

PCO 1

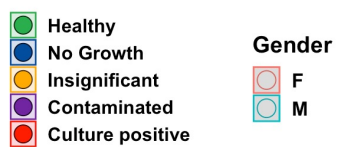**b**

PCO 2

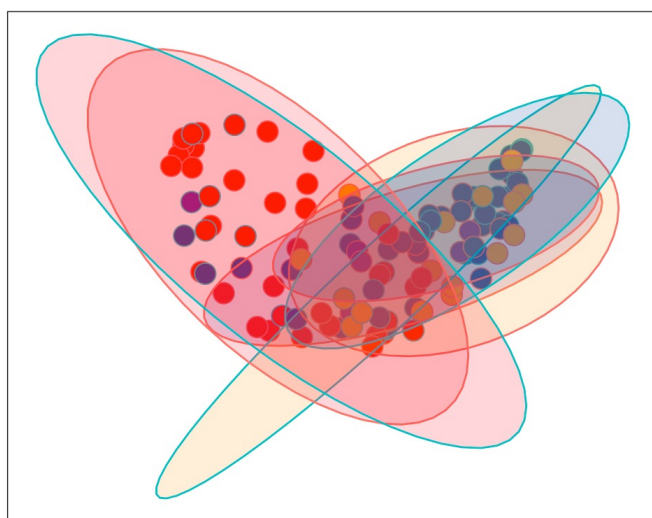

Functions: Bray-Curtis

PCO 1

Supplement: Supplementary file 3 — Additional file 3: Figure S2. Functional profile of the genitourinary microbiota. Principal coordinate analysis based on functional pathway abundance determined via HUMAnN2 for a) all female participants and b) all symptomatic participants of both sexes. Individual samples are colored based on clinical categorization. [file 40168_2021_1204_MOESM3_ESM.pdf]

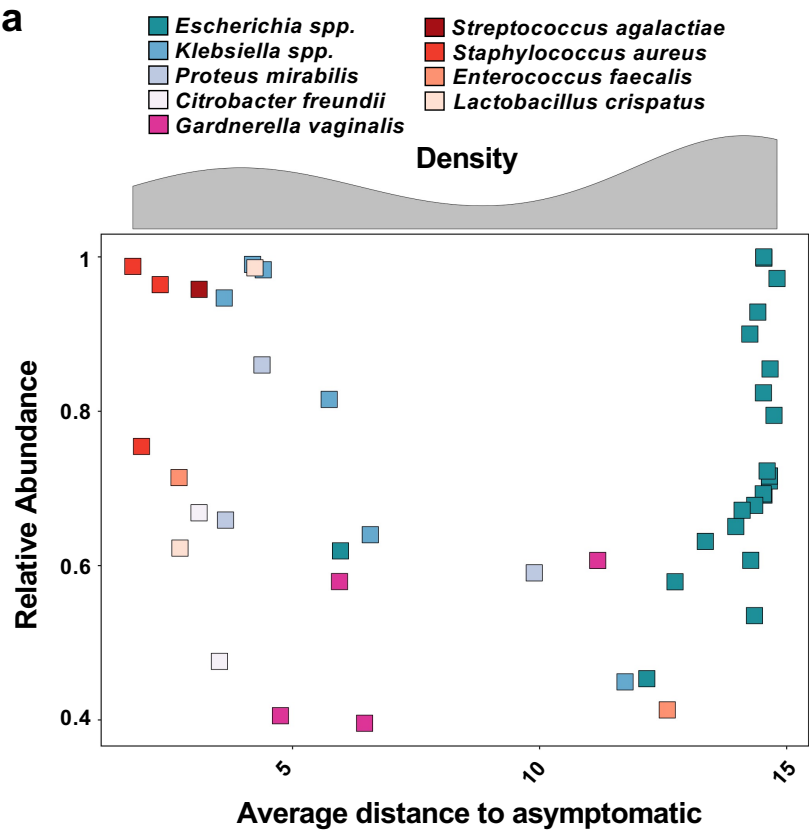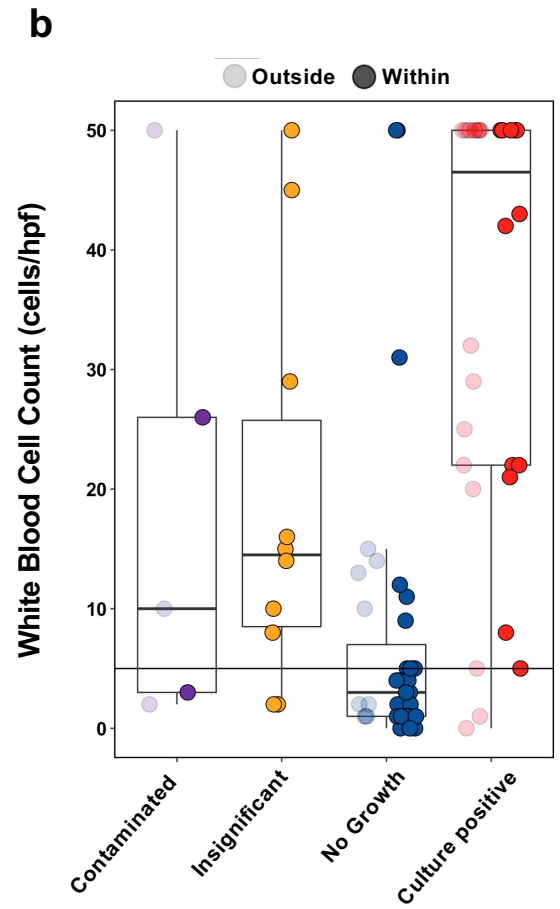

Supplement: Supplementary file 4 — Additional file 4: Figure S3. Samples outside the range of asymptomatic microbiome states are dominate by E. coli but not associated with increased inflammation. B) Scatterplot of the relative abundance of the most abundant intestinal microbiota from ‘culture-positive’ specimens plotted against each specimen’s average distance from the centroid of the asymptomatic distribution of microbiota states as depicted in Fig. 3a. Sample density at each distance is plotted on top of the graph. b) White blood cell count (cells/high power field - hpf) determined via microscopic examination in urine specimens classified into different diagnostic categories. Specimens are labeled based on whether the determined microbiota compositions fell within (dark bar) or outside (light bar) the 90% quantile of asymptomatic microbiota compositions as depicted in Fig. 3a. [file 40168_2021_1204_MOESM4_ESM.pdf]

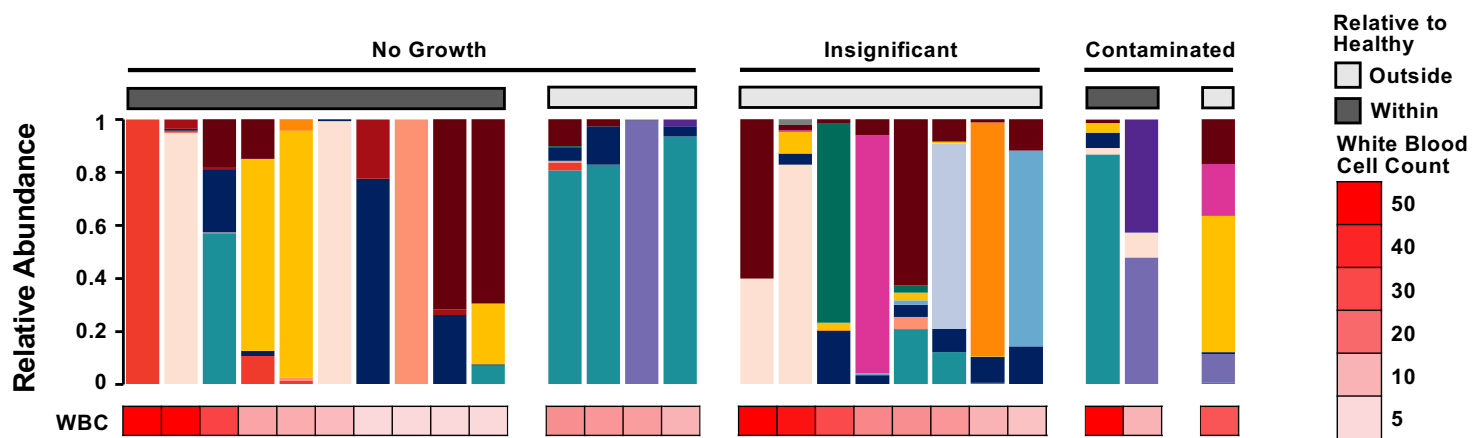

Supplement: Supplementary file 5 — Additional file 5: Figure S4. White blood cell count is independent of microbiota composition in the subset of investigate urine specimens. Microbiota composition of urine specimens (stacked bargraph) is grouped by clinical diagnostic category. Specimens are further grouped based on whether the determined microbiota compositions fell within (dark top bar) or outside (light top bar) the 90% quantile of asymptomatic microbiota compositions. Corresponding white blood cell counts determined via high power field microscopy are depicted in tiles under each bar. [file 40168_2021_1204_MOESM5_ESM.pdf]

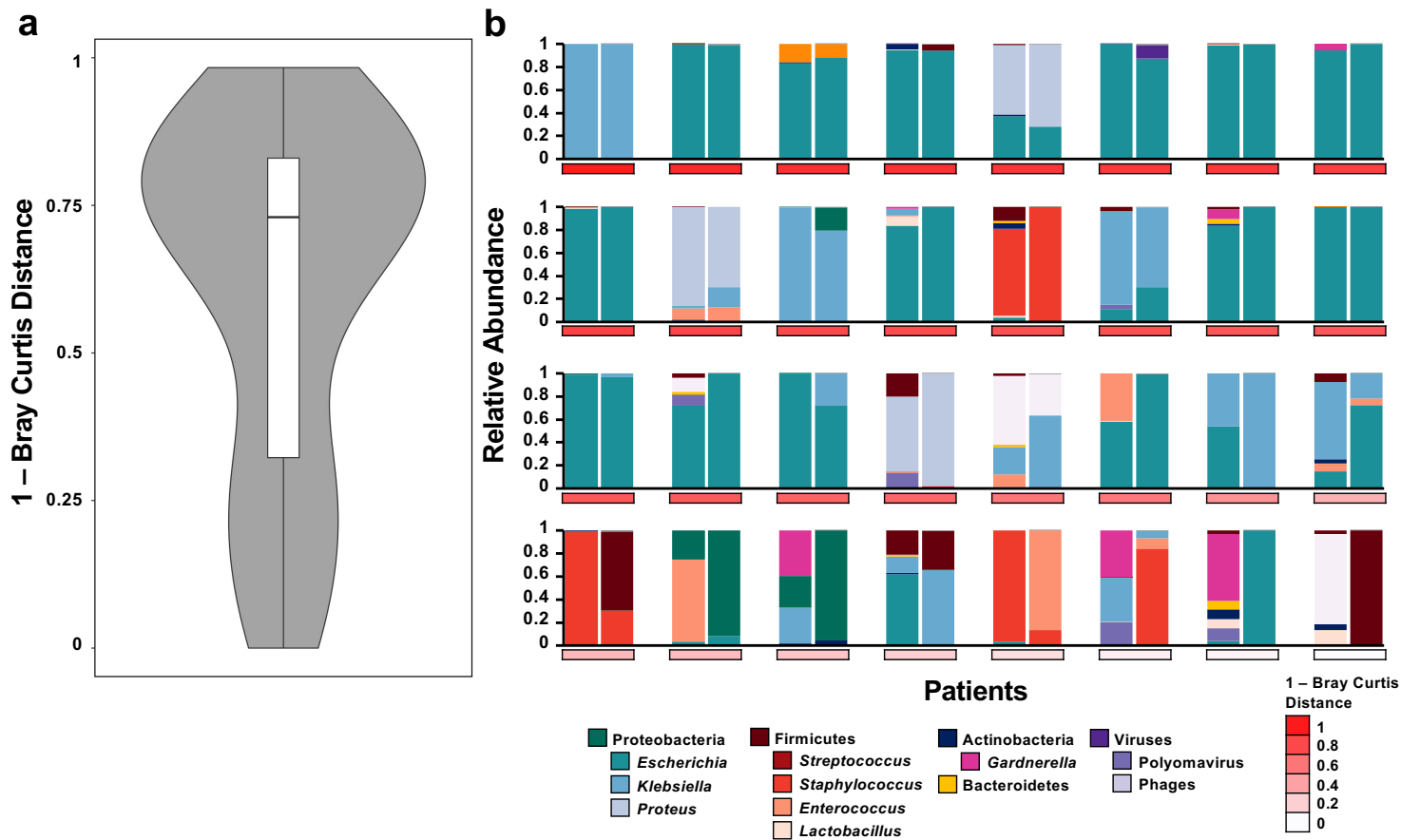

Supplement: Supplementary file 6 — Additional file 6: Figure S5. Microbiota states of individual specimens is not represented by standard-of-care urine culture. a) Bray Curtis similarity (1-Bray Curtis distance) of directly sequenced and cultured urine specimens. b) Stacked barchart depicting the microbiota composition of directly sequenced (left bar) and cultured (right bar) ‘culture-positive’ urine specimens. Pairwise Bray Curtis similarity is indicated in tiles below each sample pair. [file 40168_2021_1204_MOESM6_ESM.pdf]

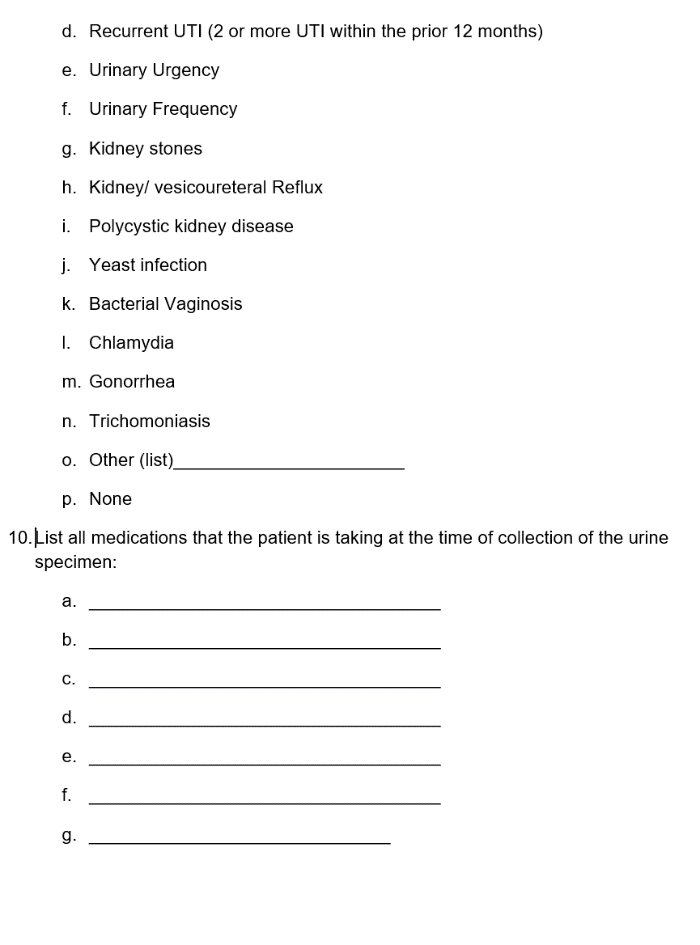

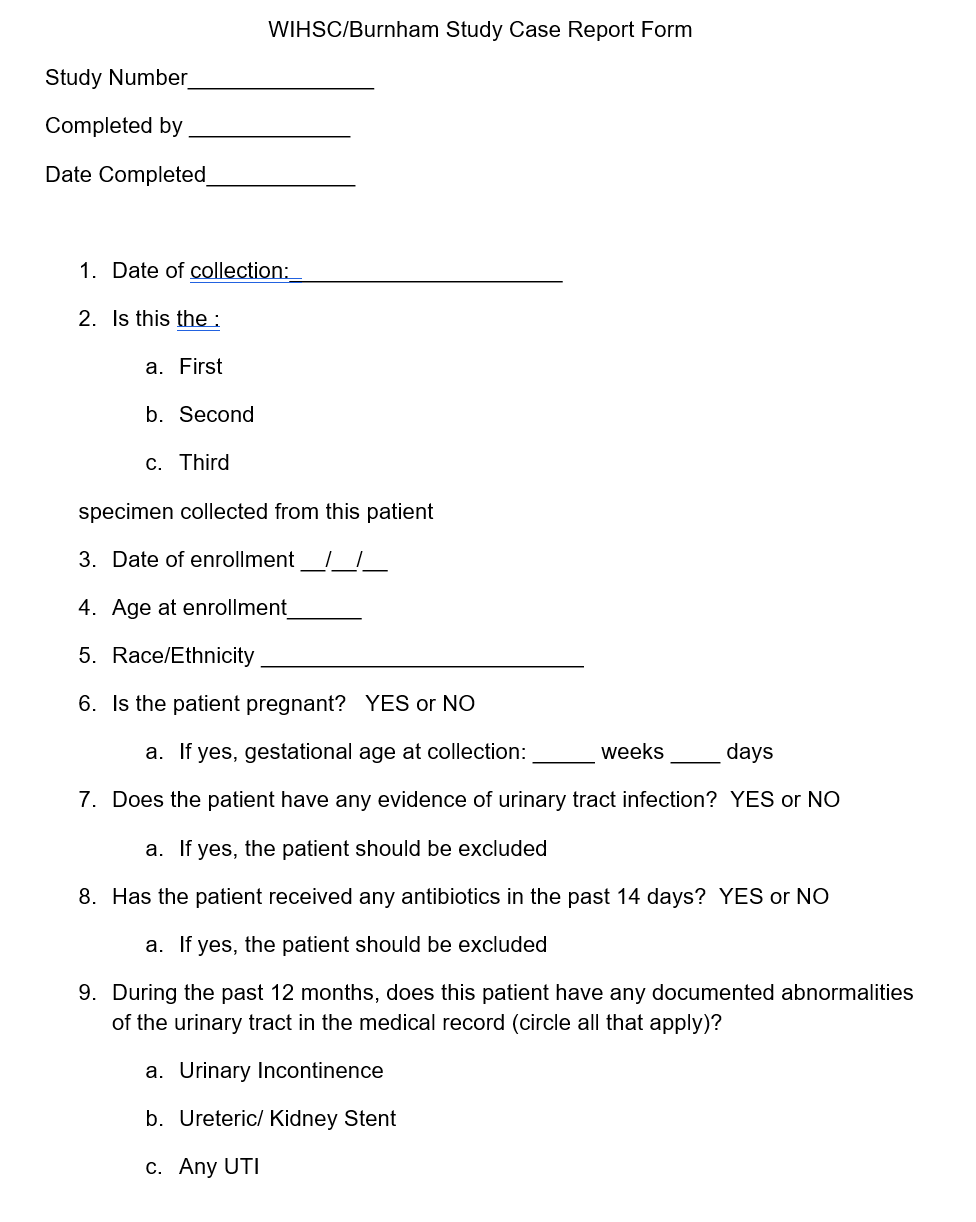


**Extended Data Table 1 | Case report form asymptomatic participants**

Supplement: Supplementary file 10 — Additional file 10. Extended Data 1 Case report form asymptomatic participants [file 40168_2021_1204_MOESM10_ESM.docx]
